# Potent Antitumor Activity of Liposomal Irinotecan in an Organoid- and CRISPR-Cas9-Based Murine Model of Gallbladder Cancer

**DOI:** 10.3390/cancers11121904

**Published:** 2019-11-29

**Authors:** Zulrahman Erlangga, Katharina Wolff, Tanja Poth, Alexander Peltzer, Sven Nahnsen, Steffi Spielberg, Kai Timrott, Norman Woller, Florian Kühnel, Michael P. Manns, Anna Saborowski, Arndt Vogel, Michael Saborowski

**Affiliations:** 1 Department of Gastroenterology, Hepatology and Endocrinology, Hannover Medical School, 30625 Hannover, Germany; erlangga.zulrahman@mh-hannover.de (Z.E.); wolff.katharina@mh-hannover.de (K.W.); spielberg.steffi@mh-hannover.de (S.S.); nwoller@web.de (N.W.); Kuehnel.Florian@mh-hannover.de (F.K.); Manns.Michael@mh-hannover.de (M.P.M.); saborowski.anna@mh-hannover.de (A.S.); 2 Department of Pathology, University Hospital Heidelberg, 69120 Heidelberg, Germany; tanja.poth@med.uni-heidelberg.de; 3 Quantitative Biology Center (QBiC), Eberhard Karls Universität Tübingen, 72074 Tübingen, Germany; alexander.peltzer@qbic.uni-tuebingen.de (A.P.); sven.nahnsen@uni-tuebingen.de (S.N.); 4 Department of General-, Visceral and Transplantation Surgery, Hannover Medical School, 30625 Hannover, Germany; Timrott.Kai@mh-hannover.de

**Keywords:** organoids, gallbladder, CRISPR/Cas9, Nal-IRI, mouse model

## Abstract

Gallbladder cancer is associated with a dismal prognosis, and accurate in vivo models will be elemental to improve our understanding of this deadly disease and develop better treatment options. We have generated a transplantation-based murine model for gallbladder cancer that histologically mimics the human disease, including the development of distant metastasis. Murine gallbladder–derived organoids are genetically modified by either retroviral transduction or transfection with CRISPR/Cas9 encoding plasmids, thereby allowing the rapid generation of complex cancer genotypes. We characterize the model in the presence of two of the most frequent oncogenic drivers—Kras and ERBB2—and provide evidence that the tumor histology is highly dependent on the driver oncogene. Further, we demonstrate the utility of the model for the preclinical assessment of novel therapeutic approaches by showing that liposomal Irinotecan (Nal-IRI) is retained in tumor cells and significantly prolongs the survival of gallbladder cancer–bearing mice compared to conventional irinotecan.

## 1. Introduction

Gallbladder cancer (GBC) is the most common biliary tract cancer and ranks sixth of all gastrointestinal cancers. In 2018, GBC is predicted to reach more than 200,000 new cases, with 165,087 cancer-related deaths worldwide [1,2]. Notably, significant differences in GBC incidence are reported among different geographical regions and ethnicities, with highest rates in South America [3]. These differences may in part be attributed to the prevalence of known risk factors that predispose to the development of GBC, such as the presence of gallstones, chronic bacterial infection (e.g. salmonella), or anomalies of the pancreatobiliary duct junction [4,5]. The median survival of GBC worldwide is low, ranging from 5.7 months to 12.89 months [6,7]. Surgical resection can improve the five year survival rate, but less than 40% of patients are amenable for surgical resection [8]. Based on the results from the ABC-02 trial published in 2010, combination chemotherapy with gemcitabine and cisplatin remains the standard of care for the treatment of patients with cancers of the biliary tract, including GBC, leading to a median overall survival of 11.7 months with a median progression free survival of eight months [9]. No established second line concepts exist, but recently presented results from the first prospective randomized phase III trial (ABC-06) provide initial evidence that patients with tumor progression under first-line CT can benefit from folinic acid, fluorouracil and oxaliplatin (FOLFOX) chemotherapy in the second line setting [10]. However, to date, no therapeutic regimen has achieved long-term disease control in GBC. Among others, a novel liposomal formulation of irinotecan (Nal-IRI), which has demonstrated superior performance in patients with advanced pancreatic cancer in combination with leucovorin and 5-fluorouracil [11], is currently being assessed in clinical studies in combination with 5-FU for first- and second-line treatment of biliary tract cancers, including GBC (1st line: NIFE [12], second-line NaliriCC [13]). Irinotecan, one of the most prevalent topoisomerase inhibitors, is a prodrug also known as CPT-11, which undergoes enzymatic activation to its active metabolite SN-38 through the action of carboxylesterases [14]. The liposomal formulation of irinotecan changes the pharmacologic characteristics of irinotecan and has been shown to have superior anti-tumor activity compared to conventional irinotecan in mouse xenograft models [15,16].

Apart from “classical” chemotherapeutic regimens, the potential value of precision oncology is increasingly recognized. Thus far, several oncogenic driver mutations have been identified in patients with gallbladder cancer, including frequent inactivating mutations in tumor suppressor genes like *TP53*, *ARID1A*, and *SMAD4,* as well as activating mutations in the *KRAS* gene. Recurrent amplifications or activating mutations in members of the ERBB2 pathway (*EGFR*, *ERBB2*, *ERBB3*, and *ERBB4* and their downstream targets) point toward a decisive role of this pathway in gallbladder carcinogenesis [17,18,19,20]. Overall, the molecular landscape of gallbladder carcinoma is heterogeneous, and the consequences of specific genetic aberrations alone or in the context of the co-mutational spectrum remains largely elusive.

In order to functionally annotate the mutational landscape of GBC and to facilitate meaningful pre- and co-clinical trials, genetically flexible in vivo models mimicking the human disease are urgently needed. Immunocompetent in vivo systems serve as a preclinical platform to assess the therapeutic efficacy and characterize the pharmacodynamic properties of novel systemic therapeutic approaches within a complex environment. An existing traditional transgenic mouse model for GBC relies on gallbladder directed overexpression of rat ERBB2. While this model recapitulates several relevant histological features of human GBC, the integration of additional alleles or other driver oncogenes requires time consuming breeding of mice [21].

In this study, we use murine gallbladder organoids to generate a genetically flexible model that allows the study of gallbladder carcinogenesis in the presence of an intact immune system. We show that expression of mutant Kras or mutant ERBB2 (ERBB2^S310F^ and ERBB2^V777L^), two of the most frequent oncogenic drivers in human GBCs, drive rapid tumor development in vivo in the presence of p53 loss. Further, we demonstrate how the model can be used to functionally validate candidate tumor suppressor genes using CRISPR/Cas9. Importantly, resulting tumors histologically resemble their human counterparts and lead to metastatic spread upon orthotopic transplantation. In order to demonstrate the utility of the model to elucidate relevant pharmacodynamic properties of novel drugs, we show that GBC bearing mice treated with Nal-IRI survive longer than mice receiving conventional irinotecan and that this effect correlates with the prolonged presence of the compound in the epithelial tumor cell compartment.

## 2. Results

### 2.1. Introduction of Cancer Drivers into GB orGanoids Leads to Tumor Formation in Mice

**Figure 1:**
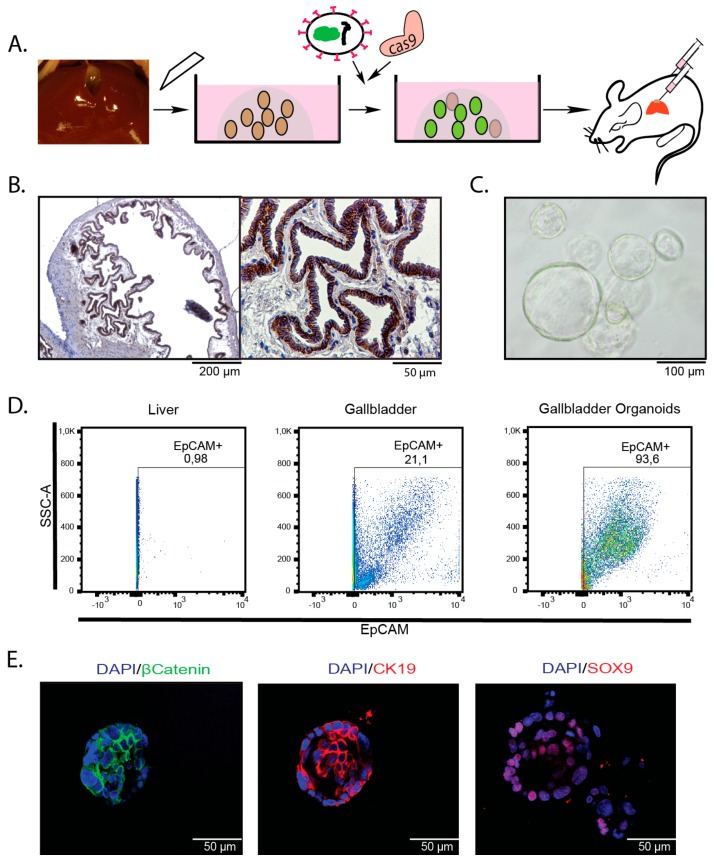
Gallbladder organoids express a biliary marker profile. (**A**) Technical outline: organoids were isolated from the gallbladders of adult mice, expanded in Matrigel, and genetically modified using CRISPR/Cas9 or by retroviral introduction of cDNAs. Genetically altered organoids were transplanted into recipient mice, either s.c. or orthotopically into the gallbladder. (**B**) Immunohistochemistry (IHC) confirms EpCAM expression within the epithelial layer of adult murine gallbladders. (**C**) Brightfield image of gallbladder organoids. (**D**) Flow cytometry analysis for EpCAM on single cell suspensions from adult mouse liver (left column), adult mouse gallbladder (middle column) and gallbladder organoids (right column). (**E**) Immunofluorescence on gallbladder organoids confirms expression of β-catenin (left), CK19 (middle), and SOX9 (right).

To assess whether gallbladder organoids can be used to study gallbladder carcinogenesis in vivo, we isolated organoids from whole murine gallbladders (Figure 1A–C). As expected, these cells express markers of biliary differentiation, such CK19, Sox9, and EpCAM (Figure 1D,E). Considering that EpCAM is uniformly expressed by the epithelial cells lining the luminal site of the gallbladder, it appears likely that the cell of origin of gallbladder organoids resides within this compartment (Figure 1B).

**Figure 2:**
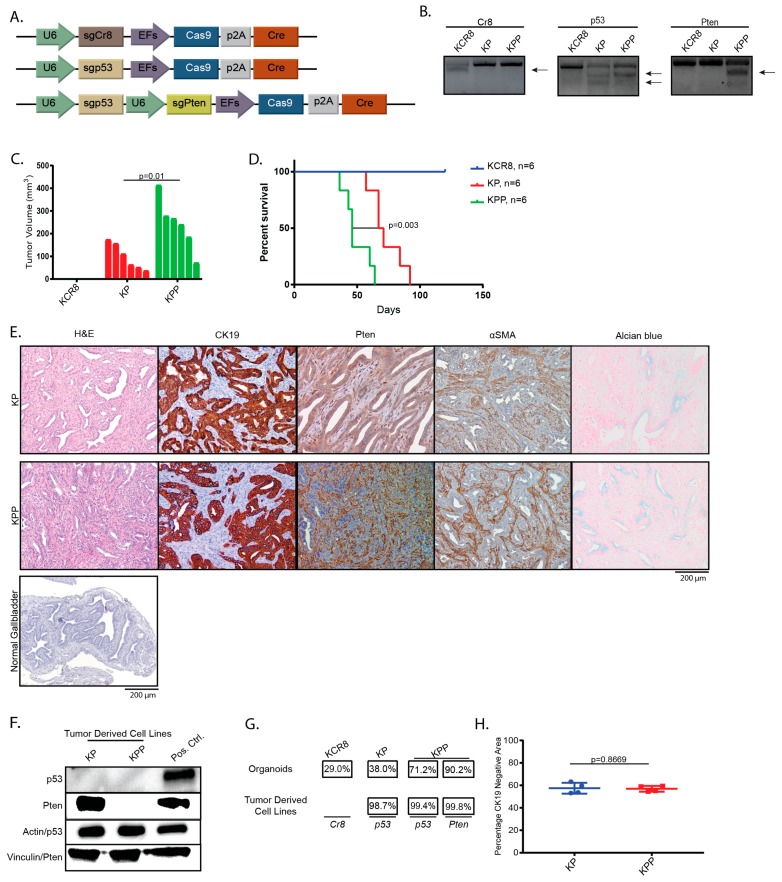
Genetically modified gallbladder organoids can give rise to gallbladder cancer (GBC) that resembles the human disease. (**A**) Schematic of plasmids used to transfect gallbladder organoids. Plasmids contain Cre recombinase, Cas9, and the respective sgRNA(s). (**B**) T7 endonuclease assay confirming cleavage after transfection and selection with blasticidin, first column: KCR8 organoids, second column: KP organoids, and third column: KPP organoids; arrows indicate cleaved bands. (**C**) Tumor volume 32 days after organoid implantation. No tumor development occurred in mice transplanted with KCR8 organoids during the four-month observation period. (**D**) Kaplan-Meyer curves of mice transplanted with KCR8, KP, and KPP organoids. Transplantation with KP and KPP organoids led to rapid tumor development (median survival: 69 days and 46 days for the KP and KPP cohorts, respectively). (**E**) Histological characteristics of GBC tumors derived from KP and KPP organoids. H&E staining of both genotypes shows GBCs classified as adenocarcinomas. IHC for CK19 confirms ductal differentiation and PTEN IHC detects loss of PTEN expression in sgPten-bearing epithelial tumor cells, but not in the surrounding stroma cells. IHC for αSMA confirms the presence of cancer-associated fibroblasts. Normal gallbladder tissue (H&E) for comparison. (**F**) Loss of p53 and PTEN confirmed on tumor-derived cell lines of the respective genotypes by immunoblotting. KCR8 organoids served as positive control. (**G**) Frequency of indels in the respective loci in preinjection organoids and in tumor derived cell lines shows enrichment of p53- and PTEN alterations during tumor development. (**H**) The relative stromal content of KP and KPP derived tumors (considering CK19 negative area as a surrogate for the relative stromal content) did not differ significantly (57.44% and 56.97%, respectively; *p* > 0.8669).

*TP53* and *KRAS* are among the most frequently mutated genes in GBC [7,18]. To investigate whether alteration of these genes in gallbladder organoids leads to GBC, we first generated organoids from Kras^lslG12D^ mice. Activation of the latent *Kras* mutant and loss of *p53* with and without loss of *phosphatase and tensin homolog* (*Pten)* was achieved by co-transfecting pt3-PGK-*Blasticidin*-P2A-*EGFP* and a plasmid co-encoding Cre recombinase, Cas9, and either a single sgRNA against *p53* or two sgRNAs targeting *p53* and *Pten* (Figure 2A) [22], followed by selection with blasticidin. An sgRNA directed against a non-genic region on chromosome 8 (sgCR8) [23] served as a negative control. Efficient genome editing was confirmed after selection and expansion by T7 endonuclease assays (Figure 2B).

Following transplantation, we observed tumor growth in the Kras^G12D/wt^;sgp53 (KP) and Kras^G12D/wt^;sgp53;sgPten (KPP) cohorts, but not in animals injected with Kras^G12D/wt^;sgCR8 organoids (KCR8) (Figure 2C). Accordingly, recipient mice reached endpoint criteria with a median latency of 46 days vs. 69 days after implantation of organoids in the KPP and the KP cohort, respectively (Figure 2D).

Histological examination of the tumors in both the KP and KPP cohorts revealed mostly tubular adenocarcinomas with areas of mucin production, as assessed by Alcian blue staining (Figure 2E, Table S1). Loss of PTEN in the KPP tumor cells, but not in the recipient-derived stromal cells was confirmed by IHC on histological sections (Figure 2E). Loss of PTEN in KPP and loss of p53 in KP and KPP tumor derived cell lines was detected by western blot (Figure 2F, Figure S1). The increased frequency of indels in tumor derived cell lines compared to preinjection organoids indicates positive selection of the targeted genes *p53* and *Pten* during tumor development (Figure 2G). As expected, the majority of indels are predicted to cause frameshifts (for details see Figure S2). Since a prominent stromal reaction is a hallmark of GBC, we quantified the CK19 negative area as a surrogate for the relative contribution of the tumor stroma to the tumor volume. Approximately 57% of the tumors stained CK19 negative, with no significant differences between the KP and KPP groups (57.44% vs. 56.97%) (Figure 2H). Thus, tumor development in the KP and KPP cohorts validates the suitability of our model to generate GBCs with complex cancer genotypes in vivo using CRISPR/Cas9 that histologically resemble crucial characteristics of the human disease.

### 2.2. Tumors Derived from Orthotopic Transplantation of Genetically Altered orGanoids Frequently Metastasize to the Lung

**Figure 3:**
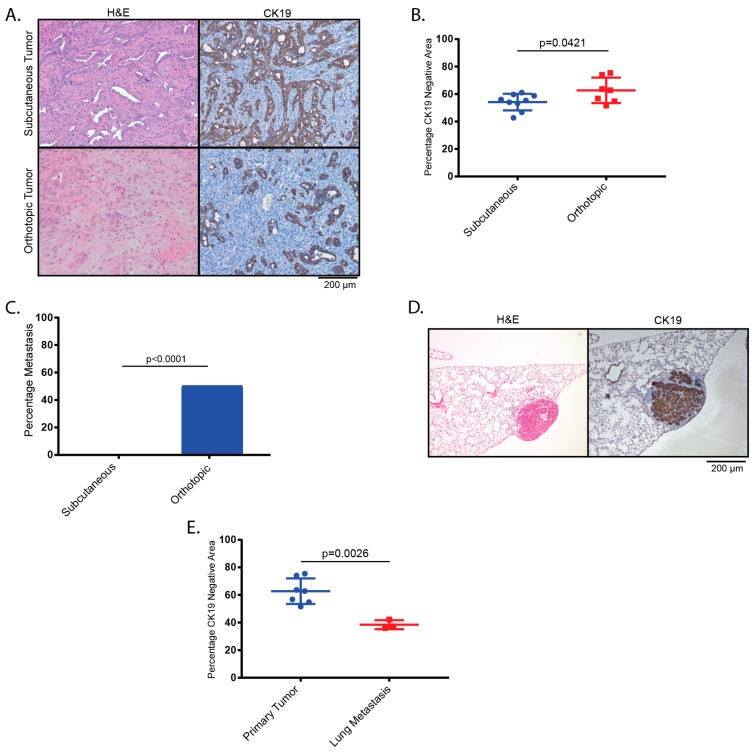
Genetically altered gallbladder organoids lead to metastasis upon orthotopic implantation. (**A**) Both s.c. and orthotopically implanted KPP organoids lead to GBCs classified as adenocarcinomas. (**B**) Orthotopic GBCs presented with a larger stromal compartment as assessed by quantification of the CK19 negative area (54.15% and 62.69%, respectively (n = 9 and n = 7, respectively, *p* = 0.0421)). (**C**) Lung metastases were present in 5/10 mice after orthotopic transplantation and in 0/10 mice after s.c. transplantation with KPP organoids. (**D**) H&E and CK19 staining of lung metastasis. (**E**) Compared to the parental orthotopic tumors, CK19 negative area as a surrogate for relative stromal content is significantly reduced in lung metastases (62.69% and 38.48% respectively, n = 7 and n = 3, respectively, *p* = 0.0026).

Next, we assessed whether the histological presentation and/or the development of metastatic spread in our murine GBC model depends on the site of implantation. KPP organoids were either injected orthotopically into the gallbladder or subcutaneously (s.c.) into the flanks of recipient mice. Histologically, tumors from both sites presented as adenocarcinomas, with a moderately increased stromal content in the orthotopic group as assessed by CK19 negative area (Figure 3A,B). Notably, lung metastatic disease was exclusively detected in 50% of orthotopically transplanted mice but in none of the mice that received flank injections (Figure 3C). Compared to the parental tumors, the metastases within the lung displayed dense aggregates of tumor cells and a significantly reduced stromal content compared to the parental tumor as assessed by CK19 negative area (Figure 3D,E).

### 2.3. Overexpression of Activating ERBB2 Mutants Give Rise to GBC

**Figure 4:**
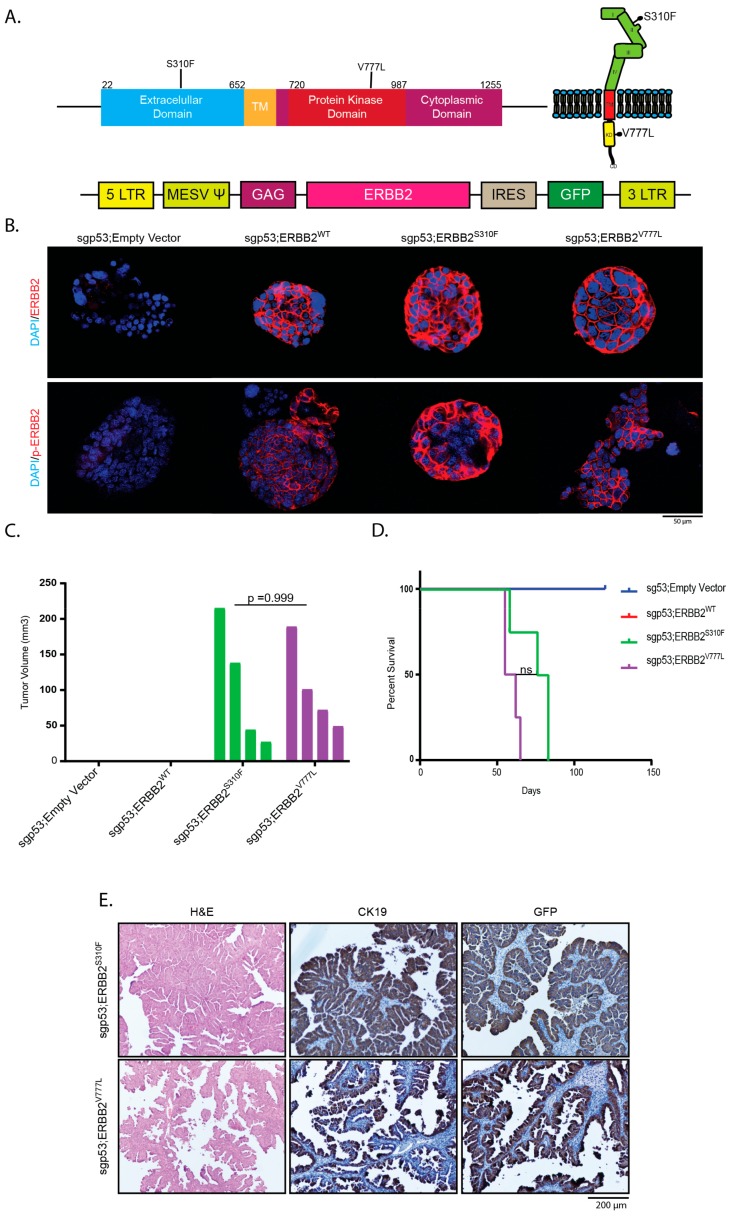
Mutant ERBB2 cooperates with loss of p53 and leads to papillary GBC in recipient mice. (**A**) Top: Schematic of human ERBB2, indicating the location of two point mutants (S310F and V777L). Bottom: retroviral vector used to transduce organoids, that had been treated with an sgp53-containing plasmid (px459) to induce loss of p53. (**B**) Immunofluorescence for ERBB2 (top) and phospho-ERBB2 (bottom) on organoids harboring the indicated genetic alterations. (**C**) Tumor volumes 36 days after s.c. implantation of the respective organoids into recipient mice. All mice transplanted with sgp53;ERBB2^S310F^- and sgp53;ERBB2^V777L^ organoids exhibited tumor development, whereas sgp53;empty vector- and sgp53;ERBB2^wildtype^ organoids did not give rise to tumors over a four-month observation period. There was no significant difference in the tumor burden of mice transplanted with sgp53;ERBB2^S310F^- and sgp53;ERBB2^V777L^ organoids (*p* = 0.999). (**D**) Mice transplanted with sgp53;ERBB2^S310F^- and sgp53;ERBB2^V777L^ organoids reached endpoint criteria with a median survival of 79.5 days and 58.5 days, respectively. (**E**) H&E and IHC for CK19 and EGFP on tumors generated with sgp53;ERBB2^S310^- and sgp53;ERBB2^V777L^ organoids.

Mutations in the *ERBB2*-gene are among the most common genetic alterations in gallbladder cancer [17,18,24,25,26]. To assess their potential as oncogenic drivers in our organoid based GBCs, we stably introduced human ERBB2 and two ERBB2 mutants (ERBB2^S310F^ and ERBB2^V777L^) by retroviral transduction into gallbladder organoids, in which p53 loss had been induced by Cas9-mediated genome editing (Figure 4A). Membranous expression of both wild-type (WT) ERBB2 and both ERBB2 mutants as well as the respective phosphorylated proteins on transduced gallbladder organoids was confirmed by immunofluorescence (Figure 4B).

Both ERBB2 mutants cooperated with p53 loss and gave rise to GBC with a median OS of 79.5 and 58.5 days for ERBB2^S310F^ and ERBB2^V777L^, respectively, whereas wildtype ERBB2 unexpectedly did not lead to tumor development within the observational period of four months (Figure 4C,D). Compared to GBCs harboring Kras^G12D^ (KP and KPP), mutant ERBB2-driven tumors displayed distinct histological characteristics. While nearly all mutant Kras-driven GBC were classified as tubular adenocarcinomas, mutant ERBB2-driven GBCs were mostly of papillary/tubulo-papillary differentiation (Figure 4E, Table S1). Both genotypes led to stromal desmoplasia (Figure 2E, Figure 4E).

In summary, we show that the model histologically recapitulates prime hallmarks of the human disease and that its histology is dependent on the driving oncogenes.

### 2.4. Antitumor Effects of Nal-IRI Correlate with Increased Intratumoral CPT-11 Concentrations

**Figure 5:**
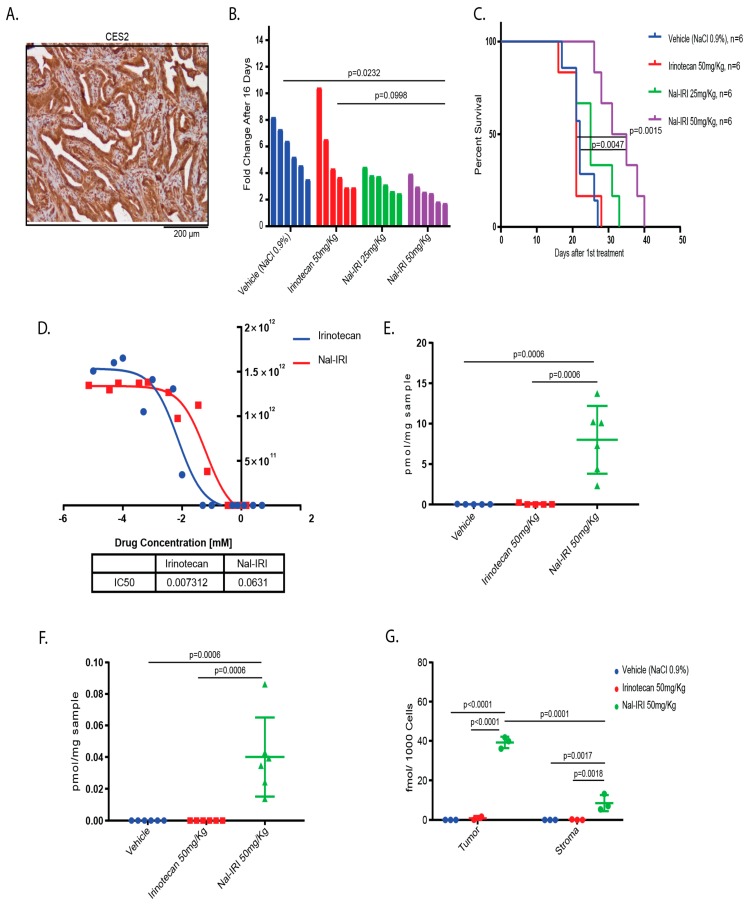
Treatment with Nal-IRI leads to improved survival in GBC bearing mice. (**A**) IHC for CES2 on a tumor derived from KPP organoids confirms CES2 expression in both the tumor cell compartment and in stromal cells. (**B**) Waterfall plots displaying growth fold changes after 16 days of vehicle, irinotecan, Nal-IRI 25mg/kg, or Nal-IRI 50mg/kg treatment. Mean fold changes are 5.87, 5.14, 3.38, and 2.60, respectively. Treatment with Nal-IRI 50mg/Kg led to significantly reduced tumor growth compared to vehicle (*p* = 0.0232). (**C**) Kaplan-Meyer curve of vehicle, irinotecan, Nal-IRI 25 mg/kg or Nal-IRI 50mg/kg treated GBC-bearing mice. Median survivals were 22 days, 21 days, 25 days, and 33 days, respectively. Nal-IRI 50mg/kg led to a significantly improved survival compared to vehicle and free irinotecan (*p* = 0.0015 and *p* = 0.0047, respectively). (**D**) IC50s of a KPP GBC-tumor derived cell line for irinotecan and Nal-IRI in vitro. (**E**) Intratumoral CPT-11 concentration of vehicle -, irinotecan - or Nal-IRI- treated, tumor-bearing mice 72 hours after a single injection with the respective drug. Treatment with Nal-IRI led to higher intratumoral CPT-11 levels than treatment with free irinotecan (*p* = 0.0006). (**F**) Intratumoral SN-38 concentrations 72 hours after a single injection with vehicle, irinotecan, or Nal-IRI. SN-38 is exclusively detectable in Nal-IRI treated mice (*p* = 0.0006). (**G**) CPT-11 concentrations within tumor- and stromal cells from vehicle, irinotecan or Nal-IRI treated tumor-bearing mice, 72 hours after a single injection. EGFP-labelled tumor cells were separated from the EGFP-negative stromal cells by FACS and subsequently analyzed with LC-MS/MS. Mean CPT-11 concentrations were significantly higher in tumor cells than in stromal cells (*p* = 0.0001) in Nal-IRI treated mice.

The topoisomerase inhibitor Nal-IRI achieved a significant increase in median overall survival of previously treated patients with pancreatic cancer [11]. Since pancreatic cancer shares several features of biliary tract cancers, such as abundant stromal desmoplasia and relative chemotherapy resistance, we wanted to assess whether Nal-IRI leads to a survival benefit in our stroma-rich GBC model in comparison to conventional irinotecan. First, we tested whether Carboxylesterase 2 (CES2), the enzyme that catalyzes the activation of irinotecan (CPT-11) to the active compound SN-38, is expressed in our murine organoid derived GBCs. IHC confirmed that CES2 is expressed in tumor cells as well as in the stromal cell compartment, suggesting that both cellular compartments are capable of activating CPT-11 to SN-38 (Figure 5A). We transplanted KPP organoids into recipient mice and, when tumor sizes reached 150 mm^3^, animals were randomized into four treatment arms (vehicle, irinotecan 50 mg/kg, Nal-IRI 25 mg/kg and Nal-IRI 50 mg/kg). Nal-IRI administered at 50 mg/kg lead to significant reduction in tumor size and prolonged the median survival to 33 days compared to 22 days for vehicle-treated mice, 21 days for free irinotecan, and 25 days for Nal-IRI administered at 25 mg/kg (Figure 5B,C). This is in stark contrast to the in vitro situation, in which Nal-IRI exhibits higher IC50s compared to free Irinotecan (Figure 5D).

Next, we aimed to delineate whether administration of Nal-IRI leads to higher intratumoral concentrations of CPT-11 and its active metabolite, SN38, compared to free irinotecan and reaches the tumor cell compartment despite the abundant desmoplasia in mice bearing s.c. GBCs. Tumors were harvested 72 hours after a single treatment with vehicle, Nal-IRI (50 mg/kg), or irinotecan (50 mg/kg). Using liquid chromatography coupled with tandem mass spectrometry (LC-MS/MS), we quantified the levels of CPT-11 and SN-38. Three days after injection, both CPT-11 (Figure 5E) and SN-38 (Figure 5F) were significantly higher in lysates from the Nal-IRI treated GBCs than in tumor lysates from mice treated with free irinotecan.

The tumor stroma may serve as a barrier for efficient drug delivery to tumor cells and stromal cells have the potential to scavenge cytostatic drugs, thereby affecting the pharmacokinetics and pharmacodynamics of drugs [27,28]. Considering that the previous experiment was performed on whole tumor lysates, we aimed to address whether Nal-IRI is predominantly retained within the tumor cells or the stroma cell compartment. To do so, we derived tumors from KPP organoids stably transfected with an EGFP expression cassette. Then, 72 hours following a single injection of the vehicle, Nal-IRI (50 mg/kg), or irinotecan (50 mg/kg), EGFP positive tumor cells and EGFP negative stromal cells were separated by FACS, and the individual fractions were subjected to LC-MS/MS analysis. Confirming our previous results from whole tumor lysates, we detected more abundant CPT-11 in the Nal-IRI treated mice than in mice receiving conventional irinotecan in both the stromal cells and the tumor cells. Furthermore, we found significantly higher CPT-11 levels in the tumor cells as compared to the EGFP negative stromal cells (Figure 5G).

Together, the improved survival of GBC bearing mice treated with Nal-IRI over conventional irinotecan is paralleled by a prolonged presence of the active drug within the tumors, where it is predominantly retained in tumor cells and not in stromal cells. These data also illustrate that our model is particularly well suited for pharmacologic investigations due to its intact microenvironment resembling the human disease.

## 3. Discussion

Recurrent key genetic alterations in patients with GBC lead to inactivation of the tumor suppressor TP53 (47.1%), to oncogenic activation of KRAS (7.8%), or to increased signaling through various components of the ERBB pathway (36.8%). In addition, multiple other genes, such as RNF43, FBXW7, and MAP2K4, have been found to be mutated, albeit at considerably lower frequency [17].

A murine model for GBC (BK5.ERBB2 mice) exists but relies on time consuming traditional breeding [29]. Considering that cancer therapy is increasingly moving towards personalized approaches, genetically flexible model systems are also needed to adequately model more complex genetic phenotypes found in GBC patients. Here, we present a murine model for GBC that relies on key tumorigenic drivers but can be easily adapted in an individualized fashion to assess the potential influence of the co-mutational spectrum on tumorigenesis and therapy response.

Organoid cultures have been established from various murine and human tissues. These cultures allow for the propagation of both normal and malignant cells and have opened up new avenues for cancer research including screens for novel therapeutics (reviewed in reference [30]). Murine gallbladder organoids can be passaged for long periods of time, are able to undergo repeated freeze/thaw cycles and can be transplanted into syngeneic recipient mice. Using untransformed murine organoids instead of fully transformed human tumor cell lines not only allows researchers to study carcinogenesis starting from a wildtype cell but also enables them to investigate GBC development and treatment strategies in the presence of an intact immune system.

We use murine gallbladder derived organoids to demonstrate how activation of mutant *Kras* or *ERBB2* in conjunction with loss-of-function of single or multiple tumor suppressor genes reliably leads to GBC in recipient mice. Disruption of candidate tumor suppressor genes or activation of latent alleles is efficiently accomplished by transfection of CRISPR-Cas9-encoding plasmids or Cre-recombinase, respectively, while retroviral transduction facilitates the rapid introduction of cDNAs encoding wildtype or mutant proteins. We generate ortho- and heterotopic GBCs featuring the most frequent genetic alterations (p53 together with mutant KRAS, as well as p53 in conjunction with mutant ERBB2). Tumors develop with 100% penetrance and can be generated with and without loss of Pten, a gene that is inactivated in a subset of human GBCs [18,31]. Since murine *Erbb2* is known to be less oncogenic than its human counterpart, we introduced the human *ERBB2* gene [32]. Interestingly, while ERBB2 is frequently amplified in various malignancies, including breast cancer, gastric cancer, or colon cancer [33,34,35], GBCs have a substantial rate of ERBB2 mutations [17,18,24]. The ERBB2 mutants ERBB2^S310F^ and ERBB2^V777L^ used in this work are located in the extracellular domain and in the tyrosine kinase domain, respectively, and lead to enhanced downstream signaling [36,37]. Notably, in our model, tumor development only occurred in the presence of the ERBB2 mutants but not upon overexpression of the WT human ERBB2, further substantiating the notion that mutant ERBB2 is a more potent cancer driver than overexpression of WT ERBB2. This data is in line with results from experiments in breast cancer (reviewed in [38]).

Histologically, both mutant KRAS and mutant ERBB2 driven tumors resemble human GBCs. Interestingly, the mutant KRAS-driven GBCs predominantly led to adenocarcinomas with tubular structures whereas the ERBB2-driven GBC frequently showed a pronounced tubulo-papillary/papillary differentiation. Both genotypes led to stromal desmoplasia, a hallmark of GBC and an important feature since the influence of the stromal compartment on therapy resistance is increasingly recognized in pancreatobiliary cancers [39,40,41,42]. Murine models that accurately depict the histology and microenvironment of human tumors are particularly important to create an adequate preclinical in vivo situation for the testing of novel therapeutic compounds. Despite exhibiting a potent anti-tumor activity either in vitro or in tumors derived from the implantation of tumor cell lines, several chemotherapeutic compounds failed in the clinical setting [43,44]. This may, in part, result from the lack of the complex interactions of a drug with the multiple different cell types and extracellular matrices present within the tumor microenvironment.

Irinotecan is a topoisomerase inhibitor that is frequently used in combination with fluorouracil (5-FU)-based chemotherapeutic regimens. It is processed to its active metabolite SN-38 by CES-enzymes, and prompted for inactivation through the conversion to a glucuronide derivate (SN-38G) as the main excreted metabolite by UDP-glucuronyltransferases [14]. Since the activity of irinotecan is limited due to major side effects and a short half-life, liposomal delivery systems have been developed. The anti-tumoral activity of Nal-IRI has been found exceed that of free irinotecan in a mouse xenograft model of colon cancer [45]. In human patients with metastatic pancreatic cancer, Nal-IRI achieved superior overall survival in combination with fluorouracil and leucovorin [11]. Stromal desmoplasia in pancreatic cancer can act as a barrier to chemotherapeutic agents [28]. Therefore, we wanted to delineate whether Nal-IRI is capable of sufficiently penetrating the GBC stroma and to reach relevant CPT-11 and SN-38 levels within the tumor cell compartment. Since both tumors cells and stromal cells express CES2, both compartments are likely capable of generating the active metabolite SN-38.

Although the in vitro activity of Nal-IRI was lower than for free irinotecan, weekly administration of Nal-IRI 50 mg/kg lead to a significant survival benefit of tumor-bearing mice, while free irinotecan was not superior to vehicle in our model. The survival benefit may in part be due to the extended levels of CPT-11 within our GBCs, as we detected substantially higher levels of both CPT-11 and SN-38 in tumors of Nal-IRI treated mice 72 hours after treatment. However, we also show a relative enrichment of CPT-11 in tumor cells over stromal cells, indicating that Nal-IRI accumulates preferentially within GBC cells and suggesting that a liposomal formulation may be beneficial in stroma-rich tumors.

## 4. Materials and Methods

### 4.1. Animal Experiments

Mice were maintained under standard housing conditions with access to food and water ad libitum and a 12-hour day-night cycle. All interventions were conducted during the day cycle. Kras^lslG12D^ mice [46] were a gift from Dieter Saur (Munich, Germany). Recipient mice (C57BL/6J and NOD.Cg-*Prkdc^scid^Il2rg^tm1Wjl^*/SzJZtm (NSG), 5–8 weeks old) were purchased from the local animal facility (Hannover Medical School, Hannover, Germany). C57BL/6J mice were used as recipients for organoids derived from C57BL/6J or syngeneic Kras^lslG12D^ mice. NSG mice were used as recipients for organoids transduced with human ERBB2 proteins. Mouse experiments were approved by local authorities (the Lower Saxony State Office for Consumer Protection and Food Safety (LAVES)). Mice were harvested when they reached endpoint criteria (sign of ill health, tumor volume > 1200 mm^3^).

### 4.2. Isolation of Murine Gallbladder Organoids

Murine gallbladder organoids were isolated from adult C57BL/6J mice or Kras^lslG12D^ mice with some modifications to published protocols [47]. Briefly, the murine gallbladder was minced with a scalpel and filtered through a 100 µm mesh. After additional washes with PBS, cells were spun at 300 *g* for 5 min, resuspended in 100% Growth Factor Reduced Matrigel (Corning, NY, USA), and plated in a 24-well plate (two 50 µL droplets per well). After solidification, Matrigel droplets were overlaid with 500 µL murine liver organoid media according to published protocols [47]. For passaging, organoids were mechanically disrupted by repeated pipetting using a P200 pipette tip, followed by a 3- to 5-minute enzymatic digestion in TrypLE Express solution (Thermo Fisher, Waltham, MA, USA).

### 4.3. Tumor Cell Isolation

Organoid derived tumors were minced with a scalpel and enzymatically digested in a shaking incubator with Collagenase IV 1 mg/mL (Sigma-Aldrich, St Louis, MO, USA) in EBSS (Thermo Fisher, Waltham, MA, USA) for one hour at 37 °C. Cells were washed with PBS, spun at 300 *g*, resuspended, and plated on tissue culture dishes in Dulbecco’s modified Eagle’s medium (DMEM) (Life Technologies Limited, Paisley, UK) supplemented with 10% fetal bovine serum (FBS) and 1% penicillin-streptomycin.

### 4.4. IC50 Cell Viability Assay:

For inhibitor treatment, tumor cell lines established as 2D cultures from primary organoid-derived tumors were plated at 5000 cells per 96-well and treated with irinotecan-HCl (Aurobindo Pharma, Munich, Germany) or Nal-IRI (Onyvide, Servier, Neuilly-sur-Seine, France) for 72 h. At the indicated time points, luminescence was assessed using the CellTiter-Glo Luminescent Cell Viability Assay on a Glomax Multi Detection System (Promega, Madison, WI, USA).

### 4.5. Flow Cytometry and Cell Sorting

Single cell suspensions from murine gallbladder organoids were prepared and incubated with the primary antibody (1:100 dilution) for 30 minutes at 4 °C (Allophycocyanin-EpCAM, ThermoFisher Scientific, Waltham, MA, USA, Cat. #17-5791-80). Flow cytometry was performed on a FacsCanto (BD Biosciences, San Jose, CA, USA) and analysis was performed using Flowjo (Flowjo LCC, Ashland, Oregon, USA). For cell sorting, single cell suspensions were prepared from organoid derived tumors as described above. EGFP-positive and EGFP-negative cells were separated by fluorescence activated cells sorting (FACS) at the institutional cell sorting facility (Hannover Medical School, Hannover, Germany).

### 4.6. Subcutaneous and Orthotopic Transplantation of Organoids

For subcutaneous (s.c.) injections, 0.5 × 10^5^ organoids were resuspended in 50 µL DMEM F12/Advanced with 50% Growth Factor Reduced Matrigel (Corning, NY, USA) and injected s.c. into the rear flanks of recipient mice. For orthotopic transplantation, mice were starved for 2 h before the surgery. A substernal 5 mm longitudinal incision was performed, the gallbladder was exposed, and the bile was aspirated using a 31G syringe (BD Medical, Le Pont de Claix, France #324826). Subsequently, 0.5 × 10^5^ organoids were resuspended in 10 µL of 100% Growth Factor Reduced Matrigel (Corning, NY, USA) and were implanted using a 31G syringe (BD Medical, #324826). After retraction of the needle, the injection site was compressed with a sterile cotton swab, and the abdominal cavity was washed with 2 mL of sterile pre-warmed water. The abdominal wall was closed layer-wise using absorbable sutures.

### 4.7. Plasmids

The U6-sgRNA-Cas9-P2A-Cre plasmid was a gift from Lukas E. Dow. sgRNA against *Cr8, p53,* and *Pten* were inserted as described previously [48]. The pMSCV-*ERBB2*-IRES-*EGFP* was a gift from Martine Roussel (Addgene, Watertown, MA, USA, plasmid #91888). *ERBB2* mutants were generated via site-directed mutagenesis PCR. The sgRNA against *p53* was cloned into pX459 as described previously (Addgene, plasmid #48139) [49].

### 4.8. Transfection and Retroviral Transduction of Organoids

Gallbladder organoids derived from Kras^lslG12D^ mice were transiently cotransfected with pt3-PGK-*Blasticidin*-P2A-*EGFP* and either U6-sgCr8-EFS-Cas9-P2A-Cre (KCR8 organoids), U6-sgp53-EFS-Cas9-P2A-Cre (KP organoids) or U6-sgp53-U6-sgPten-EFS-Cas9-P2A-Cre (KPP organoids) using Lipofectamine2000 (ThermoFisher Scientific, Waltham, MA, USA) and selected with blasticidin (20 µg/mL). Prior to transduction with different ERBB2 expressing retroviruses we transfected gallbladder organoids from C57BL/6J mice with px459_sgp53 and selected with puromycin (50 µg/mL).

To mark organoids with a green fluorescent marker (EGFP), we cotransfected pt3-PGK-*Blasticidin*-P2A-*EGFP* with the sleeping beauty-13 plasmid (kindly provided by David A. Largaespada, University of Minnesota, Minneapolis, MN, USA) using Lipofectamine2000 and selected with blasticidin (20 µg/mL). MSCV-based retroviruses (pMSCV-*ERBB2*-IRES-EGFP) were produced in Platinum-E retroviral packaging cells (Cell Biolabs, San Diego, CA, USA), concentrated using Retro-X concentrator (Clontech, Mountain View, CA, USA), and supplemented with polybrene (4 µg/mL) prior to transduction of organoids.

### 4.9. T7-Endonuclease Assays and Quantification of Indel Frequency in Edited orGanoids and Tumor Derived Cell Lines

Cas9-mediated DNA cleavage with sgCr8, sgp53 and sgPten were verified using the T7 Endonuclease I EnGen Mutation Detection Kit (NEB, Ipswich, MA) according to the manufacturer’s manual. PCR products were heteroduplex annealed and treated with Endonuclease T7. Next-generation sequencing (NGS) to determine indel frequency was performed at the genomics core unit at Hannover Medical School. Target regions from genomic DNA were amplified using corresponding primers, the PCR amplicons were pooled per sample in equimolar concentrations. The sequencing fragment libraries were prepared from 50 ng DNA with the NebNext Ultra II DNA Library Prep Kit from NEB (Ipswich, MA, USA), following the manufacture’s protocols. Sequencing was performed on a MiSeq (Illumina, San Diego, CA, USA) Nano Flowcell. Indel frequency was determined by filtering the fastq reads for the target region and the 20 bp sequence surrounding the expected cleavage site of the respective sgRNA and direct counting of WT and indel reads. The analysis of editing events was performed using the ampliCan ([50]) method. We created a design table with our amplicon sequences, primers and sgRNA sequences for all five samples, together with the raw MiSeq sequencing data. With the aforementioned MiSeq data and the created design table (Table 1). we employed the ampliCanPipeline to compute summary metrics such as the number of frameshifts, and several other metrics. The analysis was performed in GNU R using a Jupyter Notebook ([51]). Downstream analysis plots for the size of observed indels were plotted using ggplot2’s violin-plot functions to describe the variance in indel sizes on a sample level appropriately. All other final results were plotted using ggplot2 ([52]) in R using a custom analysis script 

### 4.10. Immunohistochemistry, Immunofluorescence and Alcian Blue:

Paraffin-embedded tissue slides were deparaffinized and rehydrated. For Alcian-Blue staining (Serva Electrophoresis, Heidelberg, Germany) the deparaffinized slides were immersed in 3% acetic acid and stained in Fast Red for 30 seconds. Hematoxylin and eosin (H&E) staining and immunohistochemistry (IHC) were performed as described [53]. For IHC, we used the following primary antibodies: Abcam (Cambridge, UK): CK19 (ab133496), αSMA (5694) were diluted 1:250; Cell Signaling Technology (Danvers, MA, USA): PTEN XP (9188s) was diluted 1:200; Santa Cruz (Dallas, TX, USA): Ep-CAM (sc-66020) was diluted 1:200; Merck (Darmstadt, Germany): CES2 (ABS1065) was diluted 1:200. The secondary biotin-conjugated antibody (goat-anti-rabbit, #B-2770, Life Technologies, Carlsbad, CA, USA) was diluted 1:250. For immunofluorescence, Santa Cruz Biotechnology: ß-Catenin (sc-7963), CK19 (sc-33111), and Sox9 (sc-20095); Cell Signaling Technology (Danvers, MA, USA): ERBB2, (2165s); Novus Biological (Centennial, CO, USA): p-ERBB2 (NB100-81960) were all diluted 1:50.

### 4.11. Immunoblotting

Immunoblotting was performed as previously described [53]. We used the following primary antibodies: Cell Signaling Technology (Danvers, MA, USA): PTEN XP (9188S), ERBB2 (2165S), were diluted 1:1000, Vinculin XP (13901) was diluted 1:5000; Novus Biological (CO, USA): p-ERBB2 (NB100-81960) was diluted 1:1000; Leica Biosystem (Buffalo Grove, IL, USA): p53 (P53-PROTEIN-CM5) 1:1000. Secondary antibodies: Cell Signaling Technology: goat-anti-rabbit (7074S) was diluted 1:1000. For the p53 western blot organoids and tumor derived cell lines were treated with Doxorubicin (1 ng/mL) for four hours.

### 4.12. In Vivo Chemotherapy Treatment

C57Bl/6 mice were injected s.c. with KPP organoids and randomized upon detection of a tumor of 150 mm^3^ into one of three treatment arms (Irinotecan, n = 6, 50 mg/kg, intravenous, Aurobindo Pharma, Munich, Germany) or (Nal-IRI, n = 6, 25 mg/kg, intravenous, Onyvide, Servier, Neuilly-sur-Seine, France) or (Nal-IRI, n = 6, 50 mg/kg, intravenous, Onyvide, Servier, Neuilly-sur-Seine, France) or vehicle (NaCl 0.9%, n = 6) arm. Tumor growth was followed by caliper measurements and mice were harvested upon reaching endpoint criteria (tumor volume > 1200 mm^3^, signs of ill health).

To quantify the levels of SN-38 and CPT-11 in GBCs we treated mice harboring a GBC (KPP organoids) of > 500 mm^3^ with a single treatment of either vehicle, Irinotecan (50 mg/kg) or Nal-IRI (50 mg/kg). Then, 72 hours after the treatment, tumors were harvested and either analyzed directly using liquid chromatography coupled with tandem mass spectrometry (LC-MS/MS), or FACS-sorted for the EGFP-positive and EGFP-negative fractions prior to LC-MS/MS. All LC-MS/MS analyses were done at the metabolomics core facility of Hannover Medical School (MHH, Hannover, Germany).

### 4.13. Determination of CK19-Negative Area

The CK19-negative area was determined by automatic thresholding using Fiji, ImageJ (National Institutes of Health, Bethesda, MD, USA). Five non-overlapping low-magnification fields of view were assessed per tumor.

### 4.14. Statistical Analysis

Experimental data were analyzed using GraphPad Prism software. If not stated otherwise, a *p*-value of < 0.05 was considered significant. We applied a two-tailed *t*-test to compare the CK19-negative area of tumor samples. We used the Log-rank (Mantel-Cox) test to calculate differences in animal survival. A *p*-value of <0.008 was considered significant when making individual comparisons among four different cohorts of tumor bearing mice [54]. One-way ANOVA with Tukey’s Multiple Comparison Test was used to assess differences in tumor growth and also in CPT-11 and SN-38 levels. Two-way ANOVA with Bonferroni’s test was used to calculate the difference of CPT-11 and SN-38 in different treatments and compartments. Chi-square was used to calculate the proportions of lung metastasis in mice bearing s.c. and orthotopic GBCs.

## 5. Conclusions

We present and characterize an organoid-based GBC mouse model that facilitates the rapid interrogation of putative cancer genes using CRISPR-Cas9 technology. With its intact tumor microenvironment and the close histological resemblance to human tumors, the model is highly suited to address the efficacy and pharmacodynamic properties of novel therapeutic compounds. We utilize this model to show that Nal-IRI enriches in the tumor cell compartment and prolongs the survival of GBC-bearing mice compared to conventional irinotecan.

## Figures and Tables

**Table 1:** Primers.

Guide RNA Sequences	
*p53* sgRNA	CCTCGAGCTCCCTCTGAGCC
*Pten* sgRNA	GAGATCGTTAGCAGAAACAAA
*Cr8* sgRNA	GACATTTCTTTCCCCACTGG
**Primers used in T7 Endonuclease Mutation Detection Assay**	
T7 Mut PCR *p53* fwd	GCCATCTTGGGTCCTGACTT
T7 Mut PCR p*53* rev	CCCCGCAGGATTTACAGACA
T7 Mut PCR *Pten* fwd	GAGCCATTTCCATCCTGCAG
T7 Mut PCR *Pten* rev	CTAGCCGAACACTCCCTAGG
T7 Mut PCR Cr8 fwd	TAAGATGATTATCTGAATTCCTGGG
T7 Mut PCR Cr8 rev	TCTTATCCCCTGTGTTGGAA
**Primers Used in NGS**	
NGS PCR *p53* fwd	CCATAGGGGTTTGTTTGTTTGT
NGS PCR p*53* rev	CGCAGGATTTACAGACACCC
NGS PCR *Pten* fwd	GAGCCATTTCCATCCTGCAG
NGS PCR *Pten* rev	CACGATCTAGAAATGCGCCC
NGS PCR Cr8 fwd	TCTGAATTCCTGGGATGGGG
NGS PCR Cr8 rev	TGTGTGGCTACCCTGTTCTT

## Supplementary Materials

The following are available online at https://www.mdpi.com/2072-6694/11/12/1904/s1, Table S1. Histological classification of organoid GBC tumors.; Figure S1. Uncropped western blot images.; Figure S2. Characterization of induced indels.
