# Supplementary material for: Potent Antitumor Activity of Liposomal Irinotecan in an Organoid- and CRISPR-Cas9-Based Murine Model of Gallbladder Cancer
Source: Cancers (Basel). 2019 Nov 29;11(12):1904. doi: 10.3390/cancers11121904 (PMC6966678; doi:10.3390/cancers11121904)
Supplement: Supplementary file 1 [file cancers-11-01904-s001.pdf]

Supplementary material

Potent antitumor activity of liposomal irinotecan in an organoid- and CRISPR-Cas9-based murine model of gallbladder cancer

Zulrahman Erlangga, Katharina Wolff, Tanja Poth, Alexander Peltzer, Sven Nahnsen, Steffi Spielberg, Kai Timrott, Norman Woller, Florian Kühnel, Michael P. Manns, Anna Saborowski, Arndt Vogel, Michael Saborowski

Genetic Background	Histology	Tubular	Tubulopapillary	Papillary
KP (1)	Adeno-Ca	X		
KP (2)	Adeno-Ca	X		
KP (3)	Adeno-Ca	X		
KP (4)	Adeno-Ca	X		
KP (5)	Adeno-Ca	X		
KP (6)	Adeno-Ca	X		
KPP (1)	Adeno-Ca	X		
KPP (2)	Adeno-Ca	X		
KPP (3)	Adeno-Ca	X		
KPP(4)	Adeno-Ca	X		
KPP (5)	Adeno-Ca	X		
KPP (6)	Adeno-Ca	X	X	
Sgp53;ERBB2^{S310F} (1)	Adeno-Ca			X
Sgp53;ERBB2^{S310F} (2)	Adeno-Ca		X	
Sgp53;ERBB2^{S310F} (3)	Adeno-Ca		X	X
Sgp53;ERBB2^{S310F} (4)	Adeno-Ca		X	
Sgp53;ERBB2^{V777L} (1)	Adeno-Ca		X	
Sgp53;ERBB2^{V777L} (2)	Adeno-Ca	X		
Sgp53;ERBB2^{V777L} (3)	Adeno-Ca	X	X	
Sgp53;ERBB2^{V777L} (4)	Adeno-Ca		X	X

Supplementary Table 1: Histopathological characteristics of organoid-derived GBCs (examination performed by an experienced veterinary pathologist (T. P.)). All GBCs were diagnosed as adenocarcinomas. While KP and KPP organoid derived tumors exhibited mostly tubular differentiation, the ERBB2 mutant tumors mostly showed tubulopapillary or papillary features.

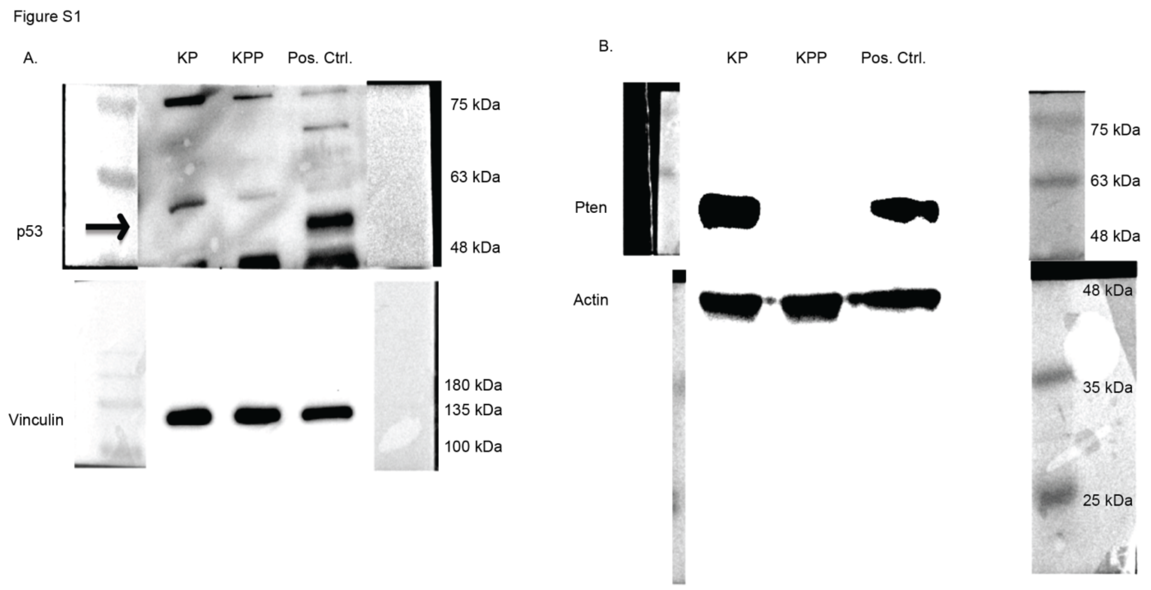

cell line	vinculin	p53
KP	17.562.004	0
KPP	18.092.175	0
Pos. Ctrl.	14.970.326	15.314.744

Cell Line	actin	pten
KP	19.534.267	34.530.539
KPP	20.609.146	0
Pos. Ctrl.	18.771.447	24.928.276

Figure S1: (A) Uncropped western blot images for p53 and loading control (Vinculin) from Figure 2F. The table shows the relative band intensities as assessed by ImageJ. (B) Uncropped western blot images for Pten and loading control (Actin) from Figure 2F. The table shows the relative band intensities as assessed by ImageJ.

Figure S2

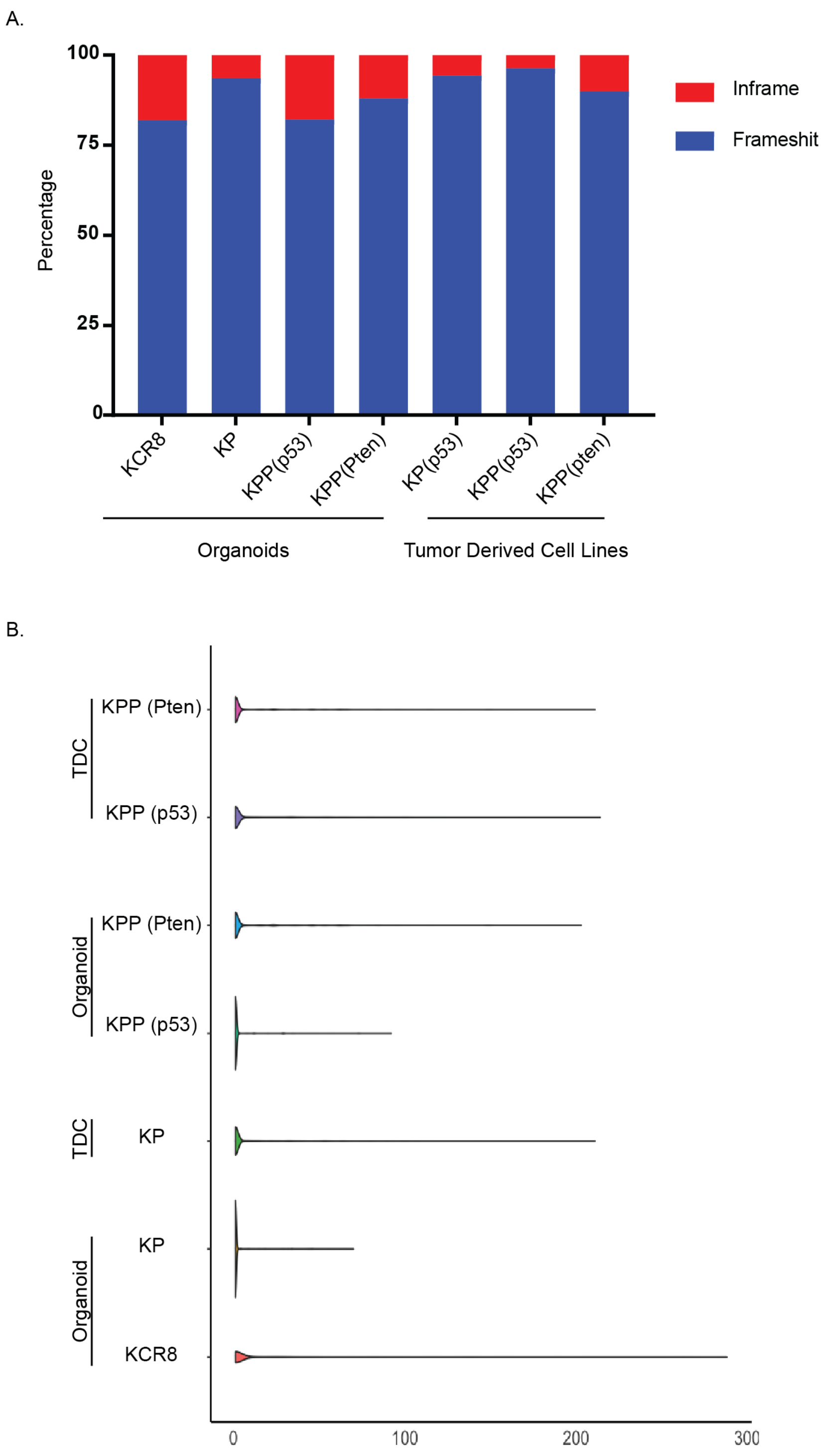

Figure S2: (A) Percentage of inframe and frameshift indels per altered reads. (B). Violin plot of the indel size assessed by *AmpliCan* (TDC – tumor derived cell line).

© 2019 by the authors. Licensee MDPI, Basel, Switzerland. This article is an open access article distributed under the terms and conditions of the Creative Commons Attribution (CC BY) license (http://creativecommons.org/licenses/by/4.0/).
